# Effects of an interdisciplinary weight loss program on fibroblast growth factor 21 and inflammatory biomarkers in women with overweight and obesity

**DOI:** 10.20945/2359-3997000000419

**Published:** 2021-11-11

**Authors:** Ana Raimunda Dâmaso, Paola Próspero Machado, Samantha Ottani Rhein, Deborah Cristina Landi Masquio, Lila Missae Oyama, Valter Tadeu Boldarine, Gabriela Iervolino de Oliveira, Lian Tock, David Thivel, Raquel Munhoz da Silveira Campos

**Affiliations:** 1 Universidade Federal de São Paulo Escola Paulista de Medicina Programa de Pós-graduação em Nutrição São Paulo SP Brasil Programa de Pós-graduação em Nutrição, Escola Paulista de Medicina, Universidade Federal de São Paulo, São Paulo, SP, Brasil; 2 Centro Universitário São Camilo São Paulo SP Brasil Centro Universitário São Camilo, São Paulo, SP, Brasil; 3 Universidade Federal de São Paulo Escola Paulista de Medicina Departamento de Fisiologia São Paulo SP Brasil Departamento de Fisiologia, Escola Paulista de Medicina, Universidade Federal de São Paulo, São Paulo, SP, Brasil; 4 UNIFESP Escola Paulista de Medicina Grupo de Estudos da Obesidade São Paulo SP Brasil Grupo de Estudos da Obesidade (GEO/UNIFESP), Escola Paulista de Medicina, São Paulo, SP, Brasil; 5 Clermont Auvergne University Laboratory of the Metabolic Adaptations to Exercise under Physiological and Pathological Conditions Clermont-Ferrand France Clermont Auvergne University, EA 3533, Laboratory of the Metabolic Adaptations to Exercise under Physiological and Pathological Conditions (AME2P), Clermont-Ferrand, France; CRNH-Auvergne, Clermont-Ferrand, France; 6 Universidade Federal de São Paulo Departamento de Biociências Santos SP Brasil Departamento de Biociências, Universidade Federal de São Paulo, Campus Baixada Santista, Santos, SP, Brasil; 7 Universidade Federal de São Paulo Programa de Pós-Graduação Interdisciplinar em Ciências da Saúde Santos SP Brasil Programa de Pós-Graduação Interdisciplinar em Ciências da Saúde, Universidade Federal de São Paulo, Campus Baixada Santista, Santos, SP, Brasil

**Keywords:** Adiposity, inflammation, weight loss

## Abstract

**Objective:**

To investigate the effects of an interdisciplinary intervention on biomarkers of inflammation and their relationship with fibroblast growth factor 21 (FGF21) concentrations in women with overweight and obesity.

**Subjects and methods:**

Thirty-one women were enrolled in a 12-week interdisciplinary weight loss program delivered by a team comprising an endocrinologist, nutritionist and exercise physiologist. Body composition; anthropometric measures; metabolic and inflammatory markers including adiponectin, leptin, and atrial natriuretic peptide (ANP) were assessed at baseline and post-therapy. The homeostasis model assessment of insulin resistance (HOMA-IR) and the homeostasis model assessment of adiponectin (HOMA-AD) were calculated. The participants were divided into two groups: those with increased FGF21, and those with decreased FGF21.

**Results:**

The sample comprised women aged 32 ± 5 years with a body mass index of 33.64 ± 3.49 kg/m^2^. Body weight, waist circumference and leptin concentration were decreased in the whole sample after therapy. However, only the group with an increase in FGF21 concentration presented significant improvements in adiponectin concentration and adiponectin/leptin ratio. Moreover, although there was a reduction of leptin in both groups, it was greater in the increased FGF21 groups. There was a reduction in ANP in the decreased FGF21 group.

**Conclusions:**

Changes in FGF21 concentrations were different among the women participating in the weight loss program, with some having increased levels and some reduced levels. Furthermore, improvements in adiponectin and the adiponectin/leptin ratio were found only in the group with increased FGF21 concentration.

## INTRODUCTION

Obesity is a chronic disease that induces a wide range of altered metabolic and inflammatory states, with a disruption of energy balance that might limit the long-term efficacy of weight loss therapy, favouring weight regain, and leading to the establishment of a vicious cycle (1). The worldwide prevalence of obesity continues to increase significantly in children, adolescents and adult populations (2,3). Moreover, obesity, especially extreme obesity, may coexist with other chronic diseases such as diabetes mellitus, hypertension, cardiovascular and non-alcoholic fatty liver diseases (4).

Interestingly, although leptin was identified more than twenty years ago as a key factor in the control of energy balance, studies have shown hyperleptinemia to be correlated with pro-inflammatory conditions in people with obesity, which may lead to the development of atherosclerosis. Inversely, adiponectin has a potent anti-inflammatory action and inhibits insulin resistance in obesity. It is important to note that both hyperleptinemia and hypoadiponectinemia are associated with obesity and metabolic syndrome in adolescents and adults (5).

Atrial natriuretic peptide (ANP) is a protein predominantly secreted by cardiomyocytes with a genetically specific structure and function, and is known for its role in the cardiovascular system. Recently, it has emerged that ANP not only has a cardiovascular role, but also acts in energy metabolism, glucose homeostasis and insulin sensitivity in organs such as adipose tissue, skeletal muscle and the liver. Evidence suggests that its role in energy dissipation is related to enhanced lipid oxidation and mitochondrial respiration in both brown and white adipose tissue, promoting increased muscular oxidative capacity. Indeed, a study demonstrated that natriuretic peptides developed anti-inflammatory actions that could promote the inhibition of inflammasome activation, including in obesity. Taken together, this evidence suggests that ANP is an important endocrine hormone that has been shown to meditate inflammatory processes and thermogenesis in clinical and experimental conditions (6-9).

Interestingly, since its discovery almost 50 years ago, fibroblast growth factor (FGF) has been shown to be a potential endocrine regulator of many physiological and pathological conditions, including diabetes, obesity, non-alcoholic fatty liver diseases and metabolic syndrome (10). Corroborating this, a previous review of publications reported that FGF21 is considered a biomarker of many metabolic diseases, and experimental studies have shown that the administration of FGF21 in obesity can reduce body weight, glucose and triglyceride, improve insulin sensitivity and increase the mass of brown adipose tissue (3,11).

Strong evidence supports the idea that FGF21 may orchestrate many actions in both physiological and pathological conditions (12,13). In the present investigation, our aim was to analyse the effects of an interdisciplinary intervention on biomarkers of energy balance and inflammatory states such as leptin, adiponectin and ANP, according to the FGF21 response in women with overweight and obesity by dividing the sample into two groups after the intervention: those with increased FGF21 and those with decreased FGF21.

## SUBJECTS AND METHODS

This study consists of a 12-week clinical interdisciplinary weight loss program delivered by a team comprising an endocrinologist, nutritionist and exercise physiologist. All procedures were in accordance with the Declaration of Helsinki and were approved by the Ethics and Research Committee of the Federal University of São Paulo (CEP n° 0305/2017; CAAE n° 66605916.3.0000.5505). All volunteers signed an informed consent form.

### Population

The present study comprised adult women (20 to 46 years old), recruited through advertisements in the media (newspapers, magazines, radio, television and social media). The sample size calculation was performed using the GPower 3.1.9.2 program with the following parameters: an effect size of 0.30, according to a previous publication (14), an observed power of 0.85, and a level of significance of 5% in respect of the analysis of variance (ANOVA two-way) model for the two groups. The sample size obtained was 28, and were separated into two groups for analysis at the end of the study: those with increased FGF21, and those with decreased FGF21.

The volunteers lived in the city of São Paulo, Brazil or nearby, so they could attend the monthly clinical interviews, and the nutritional and exercise evaluations at the university. Additionally, weekly health education information was provided to the participants via the program website on nutrition and exercise to encourage them to make long-term life-style changes.

The inclusion criteria were to present a body mass index (BMI) value ≥ 25 kg/m^2^ according to the World Health Organization (WHO) definition (15), to be female and aged between 20 and 46. The exclusion criteria were any disease that could compromise the results of the study (heart disease, musculoskeletal deformities, diseases related to the immune system, and genetic or endocrine diseases such as Cushing’s disease and Addison’s disease, identified by a physician). The major reasons for dropout were family and financial problems, followed by job opportunities.

### Clinical protocol

**Figure 1:**
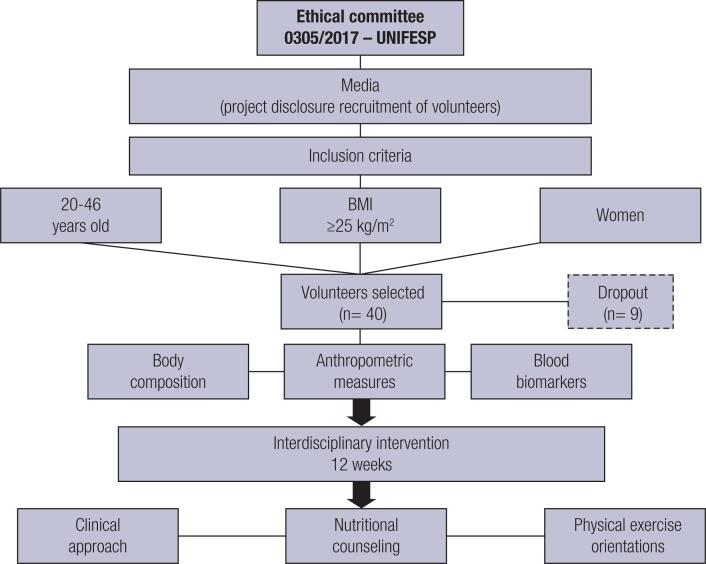
Methodological design.

The volunteers underwent two evaluations, one before and one after the intervention protocol (12th week). The evaluations comprised anthropometric measures (body weight, BMI, and circumferences – neck, waist, abdomen and hip); body composition; and blood samples were collected to analyze glucose metabolism and the inflammatory biomarkers leptin, adiponectin, ANP and FGF21. In addition, during the treatment, the volunteers received clinical support in the University from the interdisciplinary team to deal with any problems or doubts, and provide individual and group orientations (1st, 6th, 12th weeks) (Figure 1).

It is important to highlight that during the evaluations, questionnaires were applied (data not shown) that could identify some symptoms of problems commonly associated with obesity (such as depression symptoms, and body image concerns) and if a need for psychological counselling was identified by the study team, it was recommended to the volunteer.

### Anthropometric measures

Body mass was measured using light clothes and barefoot on a Filizola^®^ scale accurate to 0.1 kg and with a capacity of 180 kg. Height was measured using a wall-mounted height board (Sanny^®^) to the nearest 0.1 cm. BMI was then calculated as body mass/height^2^. Neck, waist, abdominal and hip circumferences were measured with a flexible, inelastic tape (16).

### Body composition

Body composition and basal metabolic rate were measured using a Biodynamics 310e^®^ bio-impedance meter (TBW, Sao Paulo, Brazil).

### Serum analysis

Blood samples were collected at the outpatient clinic at approximately 8:00 a.m. after an overnight fast (12 h). Glucose and insulin concentrations were assessed using commercial kits. Insulin resistance was assessed with the homeostasis model of assessment of insulin resistance (HOMA-IR) calculated using the fasting blood glucose (FBG) and the immunoreactive insulin (I) as: [FBG (in milligrams per deciliter) × I (in milliunits per liter)]/405. The cutoff value determined for classifying Brazilian subjects with insulin resistance is a HOMA-IR > 2.71 (17). The homeostasis model assessment-adiponectin (HOMA-AD) was calculated as: [FBG (mg/dL) × I (mU/L)]/[405 × Adiponectin concentration]. The assays of human FGF21 (BioVendor, Brno, Czech Republic, RD191108200R), adiponectin concentration (BioVendor, Brno, Czech Republic, RD195023100), ANP (Human NT-ProANP DuoSet DY8247-05 - R&D SYSTEMS), and leptin concentration (BioVendor, Brno, Czech Republic, RD191001100) were determined by ELISA.

### Interdisciplinary approach

#### Clinical therapy

The medical endocrinologist took an initial clinical history, and carried out a physical examination of blood pressure, cardiac frequency and body composition. In-clinic medical monitoring took place at baseline and at the end of the interdisciplinary therapy.

#### Nutritional therapy

At baseline, an individual nutritional consultation was carried out for behavioral nutritional analysis and to provide nutritional guidelines (18,19). No pharmacotherapies, antioxidants and supplements were recommended. Each participant completed a three-day dietary record at baseline and at the end of therapy. These dietary data were transferred to a computer by the same nutritionist, and the nutrient composition was analyzed using the PC program Diet Smart (DietSmart, Copyright^©^, 2012-2018) – based on Western and local food tables.

During the study, the volunteers participated in behavioral and motivational sessions of health education, with the nutritionists focused on the behavioral analysis of the diet, addressing the relationship between the individual and the food (20). Relevant themes of health nutrition were discussed including: 1) the principles of healthy eating; 2) differences between hunger and satiety and the importance of understanding these sensations in body weight control; 3) phases of weight loss – characteristics and fundamentals; 4) food behavior and nutrition; and 5) guidelines for achieving continuous weight loss and weight maintenance.

Once a week, volunteers also received videos with themes about nutrition and weight loss and health educational information aimed at improving eating habits and making changes to their lifestyle. Moreover, during the 12-weeks, the volunteers participated in five educational health sessions in groups, distributed throughout the clinical period, each lasting 60 minutes, conducted by the responsible nutritionist. In these interventions, themes related to nutritional practices were discussed and doubts raised by the volunteers were clarified. In addition, the group experience was a strategy to motivate volunteers during the program.

#### Physical activity

During the interdisciplinary therapy, the volunteers took part in five group sessions led by an exercise physiologist. Additionally, they received weekly videos with examples of exercise and health education information aimed at promoting lifestyle changes (>150 min/week). The videos and groups sessions discussed themes of 1) awareness of physical exercise; 2) practice of exercise in parks; 3) walking and running techniques; 4) the importance of setting goals; and 5) recreational physical exercise.

In addition, the exercise physiologist provided information regarding the frequency, duration and kind of physical exercise that should be undertaken, as well as recommending the monitoring of heart rate. The program included exercises to develop endurance, resistance, flexibility and balance. The Borg Scale was used by the volunteers during the exercise practice to monitor training intensity (21). Body composition variables and basal metabolic rate were used by the physiologist to recommend individual exercise training programs. The exercise modality was chosen by the volunteers according to their preferences and the recommendations of the physiologist. The program followed the recommendations of the American College of Sports Medicine (22,23).

#### Complementary health education strategies to change long-term lifestyle

The volunteers were encouraged to make lifestyle changes that would promote their health by all the professionals in their respective domains of expertise. In addition, health education videos were provided to support changes in lifestyle, with subjects that included: 1) nutrition, physical exercise and motivation – the pillars of healthy weight loss; 2) how a healthier lifestyle can improve the way you look and feel; 3) how the expression of genes responsible for obesity can be increased by a lack of exercise – sedentary behavior can increase expression of a gene responsible for obesity; 4) how to choose exercises that suit you; 5) resistance exercise, as well as aerobic exercise, can help you to lose weight; 6) how to make healthy eating choices; 7) how to use the food pyramid in your favor; 8) why should I eat every three hours?; 9) learn how to assemble dishes by combining foods efficiently; 10) slow chewing is one of the steps to weight loss success; and 11) the importance of drinking water as part of your daily diet (20,24).

### Statistical analysis

Statistical analysis was performed using the program Statistica version 7.0 for Windows. The adopted significance value was **α** ≤ 5 %. Data normality was verified using the Shapiro-Wilk test and variable values were expressed as mean ± SD. Z-score were used for non-parametric data. Analysis of variance (ANOVA two-way) was applied to compare the means between the groups, followed by Fisher’s post hoc test to identify the possible differences between groups. To analyze the effects of the intervention in the total sample, the T-test for dependent sample was applied (Table 1). The deltas values (Δ) were calculated as the differences after the intervention compared with baseline values (Δ = after intervention value – baseline value) and to compare the deltas between groups the T-test for independent groups was applied (Table 2). Correlations analyses were performed using Pearson’s test. The two groups were assigned according to the FGF21 response considering the values of the ∆FGF21; positive values were considered in respect of increased FGF21 and negative values in respect of decreased FGF21.

**Table 1:** Analysis the effects of interdisciplinary therapy on anthropometric and metabolic profile according FGF21 response

	All sample (n = 31)		Increased FGF21 group (n = 16)		decreased FGF21 group (n=15)	
	Baseline	After intervention	Baseline	After intervention	Baseline	After intervention
Body weight (kg)	89.73 ± 9.02	86.54 ± 9.04^a^	88.35 ± 8.84	85.67 ± 8.78^a^	91.20 ± 9.27	87.46 ± 9.53^a^
Body mass index (kg/m^2^)*	33.64 ± 3.49	32.44 ± 3.48	32.61 ± 3.97	31.62 ± 3.88	34.74 ± 2.60	33.31 ± 2.87
Neck circumference (cm)	35.94 ± 1.53	35.07 ± 1.39^a^	35.78 ± 1.49	34.84 ± 1.28^a^	36.11 ± 1.60	35.33 ± 1.52^a^
Waist circumference (cm)	93.91 ± 7.14	90.10 ± 6.12^a^	91.78 ± 6.53	88.39 ± 6.19^a^	96.19 ± 7.26	92.08 ± 5.62^a^
Abdominal circumference (cm)	106.93 ± 8.14	103.19 ± 7.51^a^	105.79 ± 8.17	101.51 ± 7.13^a^	108.13 ± 8.21	105.14 ± 7.74^a^
Hip circumference (cm)	120.22 ± 8.03	117.34 ± 7.93^a^	119.22 ± 8.85	117.01 ± 8.50	121.29 ± 7.20	117.72 ± 7.54^a^
Body fat mass (%)*	37.44 ± 4.37	35.75 ± 4.52	36.74 ± 4.57	35.12 ± 4.75	38.17 ± 4.17	36.43 ± 4.32
Body lean mass (kg)*	56.48 ± 4.86	55.72 ± 4.55	56.73 ± 5.39	55.70 ± 5.07	56.22 ± 4.39	55.73 ± 4.16
Glucose (mg/dL)*	93.10 ± 10.61	93.52 ± 7.15	89.75 ± 6.94	92.25 ± 6.74	96.67 ± 12.77^b^	94.87 ± 7.56
Insulin (μU/mL)*	10.83 ± 4.61	10.28 ± 4.36	10.33 ± 4.12	10.45 ± 3.93	11.36 ± 5.18	10.11 ± 4.88
HOMA-IR	2.51 ± 1.23	2.38 ± 1.01	2.32 ± 1.09	2.39 ± 0.90	2.72 ± 1.37	2.37 ± 1.14
HOMA-AD	0.89 ± 0.85	0.53 ± 0.30	0.86 ± 0.80	0.50 ± 0.23	0.91 ± 0.93	0.55 ± 0.35
Adiponectin (pg/mL)	3.78 ± 1.54	5.04 ± 2.04^a^	3.44 ± 1.25	5.34 ± 2.38^a^	4.11 ± 1.76	4.74 ± 1.68
Leptin (ng/mL)	51.48 ± 18.20	37.00 ± 14.22^a^	56.46 ± 17.12	36.03 ± 13.75^a^	46.50 ± 18.45	37.97 ± 15.09^a^
Adiponectin/leptin	0.08 ± 0.04	0.15 ± 0.09^a^	0.06 ± 0.03	0.17 ± 0.011^a^	0.09 ± 0.05	0.14 ± 0.06
ANP (pg/mL)*	373.18 ± 290.21	349.80 ± 313.17^a^	353.34 ± 203.10	384.64 ± 363.58	391.59 ± 360.01	309.59 ± 251.34^a^
FGF21 (pg/mL)	74.32 ± 44.15	127.89 ± 151.55^a^	68.68 ± 50.11	197.86 ± 185.71^a^	80.33 ± 37.58	53.26 ± 26.49^c^

HOMA-IR: homeostasis model assessment-insulin resistance; HOMA-AD: HOMA-adiponectin; ANP: atrial natriuretic peptide; FGF21: human fibroblast growth factor 21.

a Statistical difference between baseline and after intervention condition in the same group.

b Statistical difference between groups in baseline condition.

c Statistical difference between groups in after intervention condition.

* Non-parametric data normalized by Z-score.

ANOVA *two-way* followed by Fisher post hoc test.

T-test for dependent sample.

**Table 2:** Analysis of the variables variation during interdisciplinary therapy on anthropometric and metabolic profile according FGF21 response

	All sample (n = 31)	Increased FGF21 (n = 16)	Decreased FGF21 (n = 15)
Body weight (kg)	-3.19 ± 2.99	-2.68 ± 2.61	-3.74 ± 3.35
Body mass index (kg/m^2^)	-1.20 ± 1.14	-0.99 ± 0.97	-1.43 ± 1.30
Neck circumference (cm)	-0.75 ± 0.81	-0.79 ± 0.67	-0.72 ± 0.98
Waist circumference (cm)	-3.51 ± 3.48	-3.07 ± 3.04	-4.02 ± 4.00
Abdominal circumference (cm)	-3.15 ± 3.78	-3.99 ± 3.16	-2.17 ± 4.32
Hip circumference (cm)	-2.93 ± 3.90	-2.03 ± 3.13	-3.97 ± 4.54
Body fat mass (%)	-1.68 ± 4.21	-1.63 ± 5.06	-1.74 ± 3.25
Body lean mass (kg)	-0.89 ± 3.96	-1.29 ± 5.13	-0.49 ± 2.43
Glucose (mg/dL)	0.42 ± 10.09	2.50 ± 8.74	-1.80 ± 11.23
Insulin (uIU/mL)	-0.52 ± 4.77	0.20 ± 3.30	-1.25 ± 5.92
HOMA-IR	-0.144 ± 1.24	0.06 ± 0.8	-0.35 ± 1.54
HOMA-AD	-0.35 ± 0.84	-0.36 ± 0.81	-0.35 ± 0.90
Adiponectin (pg/mL)	1.26 ± 2.11	1.90 ± 2.30	0.63 ± 1.74
Leptin (ng/mL)	-14.48 ± 13.75	-20.44 ± 14.65	-8.53 ± 10.05^d^
Adiponectin/leptin	0.07 ± 0.09	0.10 ± 0.11	0.04 ± 0.05^d^
ANP (pg/mL)	-18.40 ± 443.04	16.16 ± 436.48	-52.96 ± 464.57
FGF21 (pg/mL)	53.58 ± 140.09	129.19 ± 161.46	-27.08 ± 24.86^d^

HOMA-IR: homeostasis model assessment-insulin resistance; HOMA-AD: HOMA-adiponectin; ANP: atrial natriuretic peptide; FGF21: human fibroblast growth factor 21.

d Statistical difference between increased and decreased FGF21 groups. Comparisons between groups were performed by T-test for independent groups.

## RESULTS

### Effects of therapy in entire sample

In respect of the entire sample, there was a statistically significant reduction in body weight (p < 0.001); abdominal (p < 0.001), waist (p < 0.001), neck (p < 0.001) and hip (p < 0.001) circumferences; leptin concentration (p < 0.001) and ANP (p = 0.005). On the other hand, there were increases in adiponectin (p = 0.002), the adiponectin/leptin ratio (p < 0.001) and FGF21 (p = 0.04) (Table 1).

### Comparison between the groups

**Figure 2:**
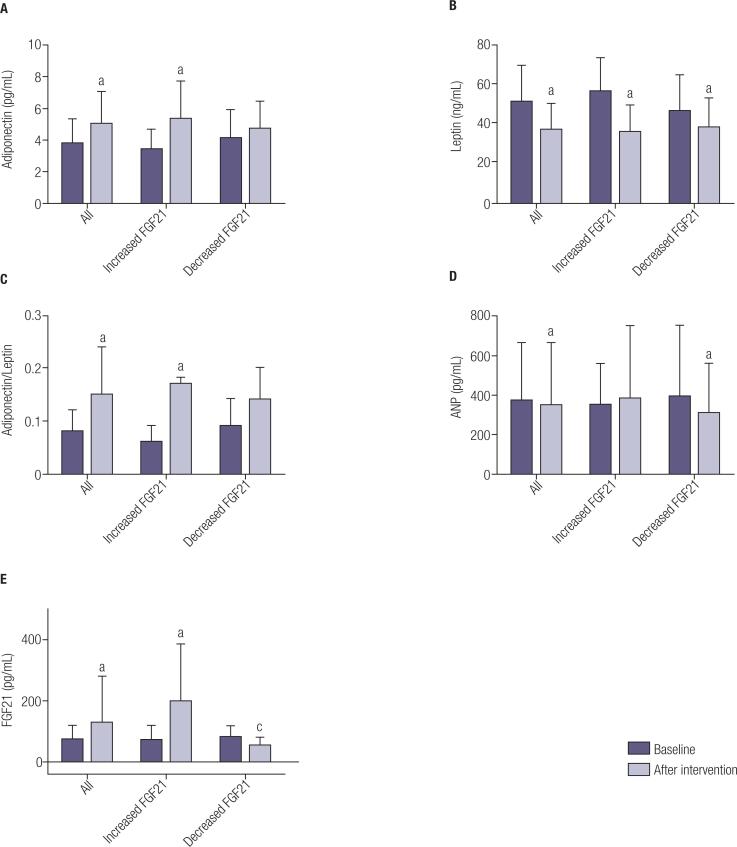
Effects of interdisciplinary intervention considering the entire sample, and the increased FGF21 and decreased FGF21 groups in respect of the variables: a) adiponectin; b) leptin; c) adiponectin/leptin ratio; d) ANP and e) FGF21. ^a^ Statistical difference between baseline and after the intervention in the same group; ^c^ Statistical difference between groups after the intervention. ANOVA *two-way* followed by Fisher post hoc test and T-test for dependent sample.

Variables in the two groups were similar at baseline: body weight (p = 0.39), BMI (p = 0.09), neck circumference (p = 0.45), waist circumference (p = 0.07), abdominal circumference (p = 0.55), hip circumference (p = 0.40), body fat mass (p = 0.38), body lean mass (p = 0.66), insulin (p = 0.51), HOMA-IR (p = 0.35), HOMA-AD (p = 0.84), adiponectin (p = 0.31), leptin (p = 0.10), adiponectin/leptin (p = 0.23), ANP (p = 0.91) and FGF-21 (p = 0.74). The only significant difference between groups at baseline was in respect of glucose (p = 0.03) (Table 1 and Figure 2A-E).

After the intervention, no differences between the groups were found in respect of body weight (p = 0.58), BMI (p = 0.16), neck circumference (p = 0.37), waist circumference (p = 0.14), abdominal circumference (p = 0.23), hip circumference (p = 0.82), body fat mass (p = 0.40), body lean mass (p = 0.98), glucose (p = 0.41), insulin (p = 0.83), HOMA-IR (p = 0.95), HOMA-AD (p = 0.55), adiponectin (p = 0.36), leptin (p = 0.74), adiponectin/leptin (p = 0.23) and ANP (p = 0.75). Only FGF21 values were different between groups after the intervention (p < 0.001) (Table 1 and Figure 2A-E).

### Effects of therapy in increased FGF21 group

There were reductions in body weight (p = 0.001), neck (p = 0.001), waist (p = 0.002) and abdominal (p < 0.001) circumferences, and in leptin concentration (p < 0.001). There were increases in adiponectin (p = 0.001), adiponectin/leptin ratio (p < 0.001) and FGF21 (p < 0.001). No differences were found for HOMA-IR, HOMA-AD and ANP (Table 1 and Figure 2A-E).

### Effects of therapy in decreased FGF21 group

There were reductions in body weight (p = 0.001), waist (p < 0.001), abdominal (p = 0.004) and hip (p < 0.001) circumferences, leptin concentration (p = 0.01) and ANP (p = 0.03). No differences were found for HOMA-IR, HOMA-AD, adiponectin and adiponectin/leptin ratio (Table 1 and Figure 2A-E).

### Changes in the metabolic profile according to FGF21 groups

Comparing the effects of therapy according to the FGF21 response, there was a greater reduction in leptin concentration (p = 0.01), and increases in adiponectin (p = 0.05) and the adiponectin/leptin ratio (p < 0.001) in the increased FGF21 group (Table 2).

### Correlation analysis

In the present investigation, it was found that ΔFGF21 correlated positively with ΔANP considering the total sample (r 0.82, p = 0.002), increased FGF21 group (r 0.97, p = 0.02) and decreased FGF21 group (r 0.73, p = 0.04). In addition, ΔFGF21 correlated positively with Δadiponectin considering the total sample (r 0.65, p = 0.02). Moreover, there was a negative correlation between ΔFGF21 and Δwaist circumference in the increased FGF21 group (r -0.98, p = 0.01).

## DISCUSSION

FGF21 is known to be a key mediator of energy homeostasis and inflammatory processes in metabolic diseases, and its concentration changes in healthy and unhealthy conditions. In the present study, the most important findings observed after weight loss therapy was that only the increased FGF21 group presented significant improvements in adiponectin concentration and adiponectin/leptin ratio. In line with these results, leptin concentration was significantly reduced in both the increased and decreased FGF21 groups. However, the increased FGF21 group, showed a greater reduction in leptin compared to the decreased FGF21 group.

The adiponectin/leptin ratio in the increased FGF21 group after weight loss therapy was almost twice that at baseline, suggesting an improvement in inflammatory state in respect of anti-inflammatory biomarkers (4,25). Although our study did not show a statistical difference after the intervention in respect of HOMA-IR, it is important to note that at baseline HOMA-IR was above cut-off values in the decreased FGF21 group but, after the intervention both groups (increased and decreased FGF-21) showed HOMA-IR values in accordance with the recommended reference values (17).

Interestingly, some experimental investigations have shown that interventions that raise circulating FGF21 levels increase insulin sensitivity and energy expenditure. In fact, it has previously been shown that FGF21 secretion by the liver may enhance blood flow and increase adiponectin secretion in the adipose tissue, increasing serum concentration. Additionally, FGF21 functions as a metabolic regulator in either an endocrine or autocrine manner in multiple organs, including blood vessels, the testes, kidneys, heart, skeletal muscle, and the brain. FGF21 acts on these organs not only by directly binding to the FGF receptors (FGFRs) of these organs in the presence of its receptor β-Klotho, but is also mediated by adiponectin or the central neural system (26,27).

Adiponectin has been studied in depth and its anti-inflammatory property is well established. This hormone is decreased in obesity and various studies from the literature show a marked increase in adiponectin concentration after weight loss therapy in adolescents and adults (28,29). FGF21 and adiponectin are among the major hormones secreted by the liver and adipose tissue, respectively (30). Mechanisms that elevate either circulating FGF21 or adiponectin have been shown to increase energy expenditure, reduce adiposity, decrease insulin resistance and hyperglycaemia, and are associated with reduced cardiovascular disease events (31).

As previously suggested by Hui and cols., both adiponectin and FGF21 are considered promising therapeutic strategies in the prevention and treatment of obesity and related comorbidities. However, the mechanism by which FGF21 promotes the expression and secretion of adiponectin is yet to be elucidated, although there is some evidence indicating that PPARγ may be at least partially responsible for this and be a missing link between FGF21 and adiponectin (32).

Surprisingly, in our study we found that individuals responded differently to weight loss in respect of FGF21 concentrations, with some volunteers showing a positive ∆FGF21 and others negative values. A recent review published by Martínez-Garza and cols. (33) highlighted the potential discrepancies observed in FGF21 concentrations, and noted that the FGF21 response can be different under stressful conditions, such as nutritional deprivation, increased physical exercise and some metabolic disorders, including obesity. Other studies have shown that carbohydrate intake, fat consumption, low protein and ketogenic diets can induce different hepatic expression of FGF21 (34-38).

In respect of physical exercise, the results are contradictory, but in general they suggest that the type, frequency, intensity and duration of exercise can influence the observed response. Previously, Morville and cols. (39) demonstrated that endurance training increase FGF21, while resistance exercise seems not to alter to its concentrations in healthy humans. Kim and cols. (40) identified higher values of serum FGF21 1h after recovery, but found no differences immediately after acute exercise, and also reported higher values of serum FGF21 after high-intensity exercise compared to mild-intensity exercise.

Therefore, our findings lead us to hypothesize that the different responses in respect of FGF21 concentrations observed in our study may have been the result of different nutritional and exercise choices of the volunteers, as the intervention protocol did not restrict the participants to one specific nutritional or exercise regime. In addition, possible mechanisms related to epigenetic factors may contribute (41), especially in respect of FGF21 resistance in obesity, however our data do not support this hypothesis.

Additionally, we observed a positive correlation between the delta of FGF21 and the delta of ANP in both analysed groups, with ANP maintaining the same concentration in the increased FGF21 group, while in the decreased FGF21 group, there was a significant reduction in ANP. These results suggest possible synergic effects of these biomarkers in the control of energy homeostasis and inflammatory processes in women with obesity, although this needs to be explored further in a larger sample with different degrees of obesity and weight-loss, as in the present investigation both groups presented similar reductions in body weight.

Interestingly, previous data support the idea that the ANP secreted by the heart in response to exercise acts on both white and brown adipose tissue, increasing the secretion of PGC1-alfa, upregulating UCP-1, and enhancing the thermogenic effects in experimental and clinical conditions (7,42). In fact, the natriuretic peptides were first associated with the control of hypertension in patients with cardiovascular diseases (43). In respect of obesity, the evidence indicates a negative correlation between atrial/brain natriuretic peptides and body weight and BMI. Different factors may be associated with this relationship, although regional variations in fat deposits, particularly in the visceral area, are frequently reported with this condition (44,45).

Additionally, the clinical interdisciplinary weight loss therapy in the present investigation favoured improvements in some biomarkers of inflammation in the whole sample, and are in accordance with the results of previous studies (25,46-48). It is important to highlight that all professionals participated in the entire research process and we believe that an interdisciplinary approach produces greater benefits for the volunteers and professionals (who were able to gain knowledge through the interactions with other professionals) when compared to isolated therapies (49).

In respect of the 12-week duration of the intervention, several other studies have reported positive results from different protocols with a similar duration, including reductions in body fat, visceral fat, body weight, BMI, lipid profile, waist circumference, leptin and adiponectin/leptin ratio (50-52). It should be noted that these robust results were found in programs that also used presential interventions that were also closely supervised by professionals.

On the other hand, semi-intensive or non-presential therapies are attractive strategies due to the lower associated costs. For example, the intervention by Turner-McGrievy and cols. (53) was based on the use of podcasts to promote weight loss and resulted in improvements in body weight, BMI, vegetable and fruit consumption and increased exercise among overweight adults. Another successful investigation, developed with overweight or obese individuals, was based on daily self-monitoring of diet and/or body weight, suggesting that this kind of intervention could serve as an effective strategy for weight loss (54). Previous studies by our group with obese adolescents found that semi-intensive therapy was effective to improve body composition, HDL-cholesterol, leptin concentration and nutritional profile, suggesting that this kind of intervention can be used to treat obesity (46,55).

A limitation of the present study is the small sample size that may have compromised the identification of possible associations among the investigated variables. However, our study contributes important data on the complex interaction between obesity and FGF21.

In conclusion, our results showed that individuals with overweight/obesity submitted to weight loss therapy demonstrated different responses in relation to FGF21 levels. Furthermore, an increase in adiponectin and adiponectin/leptin ratio were only shown in the group with increased FGF21. Future studies are necessary to investigate the mechanisms involved in the action of FGF21 in obesity, and the possible interaction with inflammatory processes and energy homeostasis.
